# Are we missing varices by implementing Baveno-VI recommendation of not screening patients with Compensated Advanced Chronic Liver Disease?

**DOI:** 10.12669/pjms.38.1.4796

**Published:** 2022

**Authors:** Faiza Sadaqat Ali, Nimrah Bader, Bader Faiyaz Zuberi, Tazeen Rasheed

**Affiliations:** 1 Faiza Sadaqat Ali, FCPS. Department of Medicine/Gastroenterology, Dow University of Health Sciences, Karachi, Pakistan; 2 Nimrah Bader, MBBS, MD. Department of Internal Medicine, University of Oklahoma Health Sciences Center, Oklahoma City, OK, USA; 3 Bader Faiyaz Zuberi, FCPS. Department of Medicine/Gastroenterology, Dow University of Health Sciences, Karachi, Pakistan; 4 Tazeen Rasheed, FCPS. Department of Medicine/Gastroenterology, Dow University of Health Sciences, Karachi, Pakistan

**Keywords:** Cirrhosis, Varices, Baveno-VI, Gastroscopy, Compensated Advanced Chronic Liver Disease

## Abstract

**Objectives:**

This study aimed to validate Baveno-VI recommendations for variceal screening in cACLD in our region and proposed our own cutoff values.

**Methods:**

Prospective cross-sectional study was conducted on cACLD patients from August 2020 till April 2021. Patients segregated into Group-A, having Liver stiffness measurement (LSM) of ≥ 20 kPa and platelet of ≤ 150 × 10^9^ cells/L; and Group-B having LSM of < 20 kPa and PLT of > 150 × 10^9^ cells/L. Gastroscopic findings were segregated into three categories, VNT, Varices Not Needing Treatment (VNNT) and No Varix (NV). ROC plots were generated for LSM and Platelet for VNT for sensitivity, specificity, Negative and Positive Predictive Values were calculated.

**Results:**

A total of 134 patients of cACLD were included. Group-A had 72 (53.7%) patients and Group-B had 62 (46.3%) patients. Group-A had 6 (8.3%) NV; 18 (25.0%) VNNT and 48 (66.7%) VNT. Group-B had 26 (41.9%) NV, 24 (38.7%) VNNT and 12 (19.4%) VNT. The sensitivity of 66.7%, specificity of 80.6% and NPV of 67.56% was obtained. Thus 19.4% VNT were missed on following Baveno VI recommendations. ROC in our study suggested cutoff value of 11.5 kPa with sensitivity of 100% and 1-sepcifity pf 78% (AUROC = 0.865; *p* < .001) of LSM below which screening gastroscopy could be avoided. The positive and negative predicted values for 84.85% and 100% respectively. Cut off value of platelet count for VNNT came out to be ≥ 97.5 × 10^9^ cells/L with AUROC 0.891 (*p* < .001), having sensitivity of 100 % and 1-specificity of 83.3%.

**Conclusions:**

Substantial number of VNT in cACLD patients are missed by following Baveno-VI recommendations and these needs to be revised on regional basis.

**List If Abbreviations:**
AASLD: American Association for Study of Liver Diseases (AASLD)
AUROC: Area Under Receiver Operating Characteristic
cACLD: Compensated Advance Chronic Liver Disease (cACLD)
CTP: Child-Turcotte-Pugh
DCLD: Decompensated Chronic Liver Disease (DCLD)
EV: Esophageal Varices
KPa: Kilo Pascal
LSM: Liver stiffness measurement (LSM)
NPV: Negative Predictive Value
NV: No Varix (NV).
PPV: Positive Predictive Value
ROC: Receiver Operating Characteristic
VNNT: Varices Not Needing Treatment (VNNT) and
VNT: Varices Needing Treatment (VNT)

## INTRODUCTION

Variceal bleeding is a major complication in cirrhotic patients that leads to substantial morbidity and mortality,^1^ and effects around 5-15% of cirrhotic patients each year. Gastroscopy is the recommended customary procedure for screening gastroesophageal varices.^2^ In order to early diagnose variceal bleeding, it is routinely suggested that every cirrhotic patient should undergo gastroscopy at the time of the diagnosis of cirrhosis.^3^

Cirrhotic patients have clinically substantial portal hypertension prior to developing Esophageal Varices (EV). It is reported that varices are found in around 70% of Child-Turcotte-Pugh (CTP) class B or C patients, and in 40% of CTP class A patients.^4^,^5^ Bearing in mind the above mentioned figures, it is evident that a significant proportion of patients with a newly diagnosed early cirrhosis undergo endoscopy needlessly. This concept became even more crucial in recent years, when cirrhosis is diagnosed earlier because of the availability of non-invasive methods for its diagnosis such as Liver Stiffness Measurement (LSM) by Fibroscan or Elastography.^6^

In 2015, at Baveno-VI consensus meeting coined the term “compensated Advanced Chronic Liver Disease” (cACLD) for early diagnosed compensated cirrhotic patients and was defined as liver stiffness measurement ≥ 10 kPa, CTP class A and absence of prior liver decompensation.^3^ In the subset of patients with cACLD, having LSM < 20 kPa and a platelet count > 150 × 10^9^ cells/L, it was recommended not to perform screening gastroscopy because of a low prevalence of Varices Needing Treatment (VNT) in this population, as compare to patients with Decompensated Chronic Liver Disease (DCLD).^3^ Transient elastography is a noninvasive method for measurement of LSM and hence clinical stratification of patients with chronic liver disease could be done on its basis. Validation of Baveno-VI criteria were performed by Maurice et al in 2016 and Silva MJ et al in 2017 and concluded that the criteria correctly identify 98% of patients who could safely avoid endoscopy.^7^,^8^ American Association for Study of Liver Diseases (AASLD) recommendations from 2017 incorporated Baveno-VI criteria and recommended that patients with LSM < 20 kPa and platelet count > 150 × 10^9^ cells/L have very low probability (< 5%) of having VNT and screening gastroscopy can be spared in these patients.^9^ In patients who do not fulfil these criteria, screening gastroscopy for the diagnosis of gastroesophageal varices is recommended when the diagnosis of cirrhosis is made.^9^

Gastroscopy negative of VNT impose a potentially avoidable financial burden on the healthcare system,^10^,^11^ as well as anxiety and poor adherence of patients because of the invasive nature of the procedure.^12^,^13^ On the one hand need for a non-invasive substitute for screening gastroscopy in cACLD is imperative, but on the other hand, Baveno-VI recommendations needs to be validated in our region. Rationale of this study is to validate and critically analyze Baveno-VI recommendation in our population and formulating guidelines for the timing of screening Gastroscopies in cirrhotic patients.

Our objective was to validate variceal screening protocol in patients with compensated advanced chronic liver disease (cACLD) as per recommendations of Baveno-VI in our region.

### Operational definition:

• ***Cirrhosis:*** If LSM by liver elastography is ≥ 12.0 kPa & FIB-4 score is ≥ 3.25.^14^,^15^
• ***Compensated Advanced Chronic Liver Disease (cACLD):*** defined by Liver Stiffness Measurement (LSM) ≥ 10 kPa, CTP class A and absence of prior liver decompensation.
• ***Baveno-VI Criteria^3^:*** Patients who have liver stiffness less than 20 kPa and a platelet count more than 150 x 10^9^ cells/L do not need to undergo screening endoscopy
• ***Varices Needing Treatment (VNT)*** defined as the presence of medium or large EV, of any size with red wale sign, or any size of gastric varices on gastroscopy.^16^
• ***Varices Not Needing Treatment (VNNT)*** Small varices without red wale sign.
• ***Grading of esophageal varices***
• ***None:*** No veins above the esophageal mucosal surface
• ***Small:*** Minimally elevated veins above the esophageal mucosal surface
• ***Medium:*** Large tortuous veins occupying < ⅓^rd^ of the lumen
• ***Large:*** Large coil-shaped veins occupying ≥ ⅓^rd^ of the lumen.

## METHODS

This prospective cross-sectional study was conducted at Dr Ruth KM Pfau Civil Hospital Karachi, Pakistan during the period August 2020 till April 2021. Both out-patients and admitted patients were selected by non-probability convenience sampling. This study was conducted in compliance with the ethical standards of the responsible institution on human subjects as well as with the Helsinki Declaration and was granted ethical approval by Institutional Review Board of Dow University of Health Sciences vide letter # IRB-1614/DUHS/Approval/2020/139. Informed consent was taken on inclusion into study. The data supporting the findings of this study are available from the corresponding author upon reasonable request.

### Sample Size:

Sample size calculation was done reported pooled Spared Endoscopy Rate (SER) of 32.8%^17^ and null hypothesis value of 50%. Both Type-I (alpha) & Type II (beta) errors were kept at 0.05. Minimum sample size was calculated as 104 patients.

### Inclusion Criteria:

All patients of compensated advance chronic liver disease, i.e., LSM ≥ 10 kPa, CTP class A and absence of prior liver decompensation from any cause, of age between 18 to 70 years undergoing screening endoscopy were included in the study. Patients were eligible for the study if they had laboratory tests performed within 1 week and LSM > 10 kPa performed within three months prior to endoscopy.

### Exclusion criteria:

History of decompensation episode(s), e.g., CTP class C cirrhosis, or CTP Class B cirrhosis with ascites/variceal hemorrhage/hepatic encephalopathy or variceal endoscopic treatment.

• Patients with limitations to undergo and/or interpret the results of TE, i.e., hepatic congestion, acute hepatitis, metabolic diseases, extrahepatic cholestasis, ascites, abdominal wound at the TE examination site, narrow intercostal spaces.
• Current hepatocellular carcinoma or other neoplasm.
• IFN-based antiviral treatment within three months (because of the possible IFN-related decrease of platelet count).
• Splenectomy
• Patients taking β-blocker.
• Any contraindication in performing gastroscopy, e.g., severe cardiopulmonary disease with desaturation (SO_2_ ≤ 90) deranged coagulation (INR ≥1.5)
• Splanchnic vein thrombosis.

### Data Collection Procedure

History was taken and recorded for demographic variables like age and gender. Laboratory work up for CBC and PT/INR was carried out in central laboratory CHK by a trained staff on automated machines. Liver stiffness measurement (LSM) was evaluated with Transient Elastography (TE) by professionally trained operator, using a Fibroscan device (Echosens, Paris, France), at the right lobe of the liver after patients fasted for at least eight hours. The M-probe was used patients with BMI ≤ 30 kg/m^2^ and XL probe was used in patients with BMI of > 30 kg/m^2^. Only cases with 08 valid measurements obtained with a success rate ≥ 60% and an interquartile range-to-median ratio ≤30% were considered valid and selected. The median valid LSM value was expressed in kPa. Grading of VNT was determined by trained endoscopists who had experience of > 2 years in Gastroscopies, at endoscopy unit CHK, and who were unaware of platelets count and Fibroscan results. All data was recorded in study proforma and entered in electronic data base.

### Group Allocation:

All Patients of cACLD fulfilling inclusion criteria were included after informed consent. Patients were segregated according to the study protocol into two Groups based on LSM and Platelets counts as Group-A having LSM of ≥20 kPa and platelet of ≤150 × 10^9^ cells/L, who according to Baveno-VI should be screened: and Group-B, having LSM of <20 kPa and PLT of >150 × 10^9^ cells/L, who according to Baveno-VI recommendation not needing screening gastroscopy. Both Groups’ endoscopic findings were segregated into 3 categories of VNT, VNNT and NV.

### Data Analysis Procedure:

Mean and standard deviation of continuous variable such as age, while frequencies and percentages (proportions) of categorical variables such as gender, were calculated. Frequencies of VNT, VNNT and NV were calculated and compared among both Groups. To generate 2x2 table VNNT and NV were merged. Validation of Baveno-VI criteria was done by identifying frequencies of VNT in both the groups, hence specificity, sensitivity, negative predictive value (NPV) and positive predictive value (PPV) of these criteria were calculated in our study patients as mentioned below. Based on our study findings, cut-off values of LSM for VNT and platelets for VNNT were obtained by plotting Receiver Operating Characteristic (ROC) curve and calculating Area Under Receiver Operating Characteristic (AUROC). Sensitivity, specificity, NPV, PPV and *p*- values were calculated. *p*- value of ≤ 0.05 was considered significant. Sensitivity, specificity, NPV and PPV were calculated by formula as under as shown in Table-I:

**Table I:** Sensitivity & specificity 2 x 2 Table.

	Group-A	Group-B
VNT +ve	True Positive (TP)	False Positive (FP)
VNT -ve	False Negative (FN)	True Negative (TN)

Sensitivity = TP/(TP+FN); Specificity = TN/(TN+FP); NPV= TN/(TN+FN); PPV= TP/(TP+FP); Missing VNT = FP in Group B, i.e., FP/(FP+TN); Spared gastroscopy = Group B/ (Group-A+ Group-B)

Statistical analysis was done using IBM SPSS Statistics for Windows, Version 26.0. (Armonk, NY: IBM Corp.). ROC plots analysis and sample size calculation was done using software MedCalc Statistical Software version 19.1.3 (MedCalc Software by, Ostend, Belgium).

## RESULTS

### Patients’ Characteristics:

A total of 134 patients fulfilling selection criteria were selected after informed consent. These included 78 (58.2 %) males and 56 (41.8%) females. Mean (SD) age of males was 36.9 (7.0) years and that of females was 36.2 (6.8) years. The etiology of liver disease was, hepatitis C in 74 (55.2%), hepatitis B in 31 (23.1%), hepatitis D in 17 (12.7%), auto-immune hepatitis in 9 (6.7%) and alcoholic liver disease in 3 (2.2%) cases.

**Fig.1:**
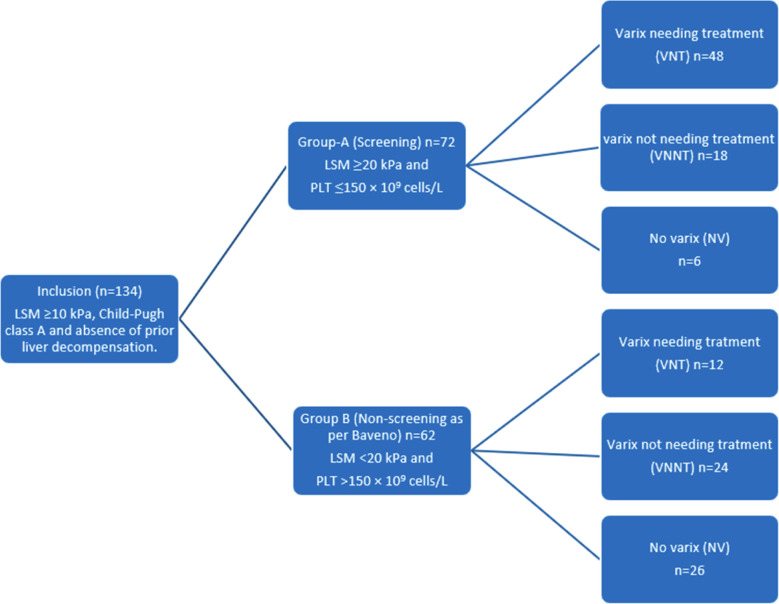


Patients were segregated into Group-A and Group-B as detailed in methodology. Group-A had 72 (53.7%) patients and Group-B had 62 (46.3%) patients. Group allocation as per protocol is given in Fig.1. Data was tested for normal distribution by Kolmogorov-Smirnov (KS) test and was found to be normally distributed. The significance levels by KS test for age was *p* = .2, for platelets and LSM in Group-A were *p* = .2 and *p* = .067 respectively and in Group-B were *p* = .187 and *p* = .2, respectively.

Comparison of age, platelet count and LSM were done between two groups by Student’s ‘t’ test and is showed no significant difference in age between two groups (*p* = .395). In Group-A platelets were significantly less (*p* < .001) and LSM was significantly high (*p* < .001) as compared to Group-B. Details are given in Table-II.

**Table II:** Age, platelets, LSM & variceal classification according to Groups.

	Age (years)	PLT^***^ Count	LSM^†^ (kPa)	Variceal Classification		

	Mean	Mean	Mean	NV^‡^	VNNT^§^	VNT^**^

				n (%)	n (%)	n (%)
Group-A (Screening) n = 72	37.08	112.71	31.49	6 (8.3)	18 (25.0)	48 (66.7)
Group-B (Non-screening) n = 62	36.06	173.55	13.39	26 (41.9)	24 (38.7)	12 (19.4)

### Validation of the Baveno-VI Criteria:

In Group-A there were 6 (8.3%) NV; 18 (25.0%) VNNT and 48 (66.7%) VNT. In Group-B there were 26 (41.9%) NV, 24 (38.7%) VNNT and 12 (19.4%) VNT. Details are given in Table-I. For sensitivity and specificity, data was converted into 2 X 2 table by merging data of NV and VNNT into patients not requiring band ligation and compared between two groups. In Group-A, 48 (66.7%) patients required EVBL while in Group-B, 12 (19.4%) patients required EVBL which were missed according to Baveno-VI recommendations. Details are in Table-III. The sensitivity (48/48+24) came out to be 66.7% while specificity (50/50+12) was at 80.6% according to our data. Positive predictive value (PPV) was 80.0% and negative predictive value (NPV) was calculated as 67.56%. As per Baveno-VI recommendations in our study, spared screening gastroscopies were 46.27% (62/134), but at the cost of missing 19.4% (12/62) of VNT.

**Table III:** Statistical comparison of age, platelets, and liver stiffness among both Groups by Student’s t-test.

	Group	Mean	SD^††^	t^‡‡^	df^§§^	*p* value
Age	Group-A	37.08	7.07	.853	132	.395
	Group-B	36.06	6.68			
PLT^***^	Group-A	112.71	13.58	-28.236	132	<.001
	Group-B	173.55	10.95			
LSM^†††^ (kPa)	Group-A	31.49	3.16	33.776	132	<.001
	Group-B	13.39	3.02			

**Table IV:** 2 X 2 Table of Groups with Variceal treatment status as per Baveno-VI in our study.

		Group-A	Group-B	Total
VNT	Count	48	12	60
	% within Group	66.7%	19.4%	44.8%
VNNT	Count	24	50	74
	% within Group	33.3%	80.6%	55.2%
Total	Count	72	62	134
	% within Group	100.0%	100.0%	100.0%

### ROC & AUROC:

**Fig.2:**
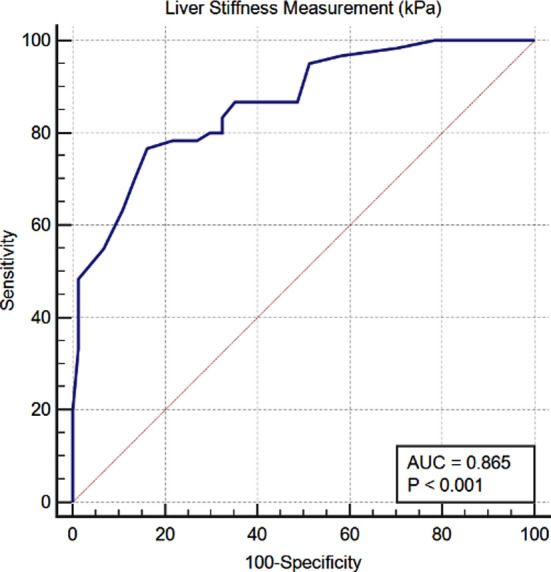


Accuracy of LSM and platelet count was evaluated according to the area under each ROC curve (AUROC). We defined the cutoff values of LSM and platelet count with aim of study to identify VNT and VNNT respectively. Corresponding to a negative predictive value (NPV) of 100%, and the main results calculated were sensitivity, specificity and positive predictive value (PPV). Cut off value of ≥ 11.5 kPa was achieved for LSM for VNT, AUROC was 0.865 (*p* < .001), having sensitivity of 100% and 1-specifity of 78% (Fig.2), rather than cut off 20 kPa as advised in Baveno-VI below which screening gastroscopy can be safely avoided. PPV 84.85 % and NPP were 100%.

Cut off value of platelet count for VNNT came out to be ≥ 97.5 × 10^9^ cells/L with AUROC 0.891 (*p* < .001), having sensitivity of 100 % and 1-specificity of 83.3% as plotted by ROC curve (Fig.3), rather than ≥ 150 × 10^9^ cells/L as in Baveno-VI. PPV 88.06 %, NPP 100%.

## DISCUSSION

We analyzed the diagnostic accuracy of Baveno-VI recommendations regarding screening endoscopies in patients with cACLD in term of specificity, sensitivity, NPV spared Gastroscopies and number of missing VNTs. We found that these recommendations made by Baveno-VI are not 100% applicable in our setup. To see missing varices needing treatment we also performed screening gastroscopies on Group B of cACLD who according to Baveno-VI recommendations should not be screened, and found that among these 62 patients, 24 (38.71%) patients still had small size esophageal varices and 12 (19.35%) patients had medium or large size esophageal varices who needed band ligation, i.e., missing VNTs, whereas 50 patients out of 62, did not have VNT, making NPV of Baveno-VI criteria 67.5%.

Although cut of value of LSM for cirrhosis varies with the etiology of CLD, in our study we did not find significant VNT in patients with LSM of ≤ 11.5 kPa, and platelet count of ≥ 97.5 × 10^9^ cells/L can be considered as cut off for early cirrhosis. With NPV of 100 % these cut off values can also be used as a non-invasive marker of avoiding screening gastroscopies. A diagnostic test accuracy meta-analysis for Baveno-VI criteria reported satisfactory outcome but of 13 studies included in that meta-analysis none was from our region and most of them were form western countries.^17^ This happened as no published data was available from our region and our report carries much importance for inclusion in policy making in future.

Some studies validated and recommended Baveno-VI criteria in their set up with high NPV but with early reassessment for worsening of these noninvasive markers. Thabut et al, in his study on 891 patients with virus-related compensated cirrhosis revealed that among the 221 patients who according to Baveno-VI criteria should not be screened, 92.8% did not have EV, 5.9% had small EV and 1.3% had medium or large varices. On the other hand, among the 670 patients who according to Baveno-VI criteria should be screened for presence of VNT, 69.6% did not have EV, 20.1% had small EV and 10.3% had medium or large varices.^6^ AASLD recommendations from 2017, demonstrated that patients with an LSM < 20 kPa and normal platelets (> 150x10^9^) carries the probability of having VNT of < 5% only, hence do not need screening endoscopy, but with early reassessment of LSM and platelet count.^3^,^9^

In cACLD patients as taken in Baveno- VI, not all patients with liver stiffness of 10 kPa have cirrhosis (this cut-off point mainly includes patients with advanced fibrosis not necessarily patients with cirrhosis). Including patients before they develop cirrhosis probably reduced the prevalence of EV, which could have falsely increased the NPV of the evaluated non-invasive criteria. Maurice et al.^10^ described in his cross-sectional study, on 310 patients with liver stiffness over 10 kPa, that Baveno-VI Consensus had sensitivity of 87%, NPV of 98%, but still missed 13% of patients with high-risk varices. Also in this study, non-cirrhotic patients were probably included, by selecting cut-off point of liver stiffness of 10 kPa, which identifies patients with advanced fibrosis and not exclusively those with cirrhosis.^18^ The authors of the study also recognized this as a limitation of getting high NPVs.^10^ Another limitation of their study was that authors did not consider Grade-I EV with red wale sign as high-risk varices, which reduces validation and the interpretation of its results.

Mattos and Mattos also highlighted his mistrust on the methodology of some studies used as a basis of the Baveno-VI Consensus and concluded that non­invasive methods “should not replace endoscopy in variceal screening at the present time”.^18^ In EASL­ALEH Clinical Practice Guidelines of noninvasive tests used for evaluating liver fibrosis and disease progression, the authors concluded that non­invasive tests cannot replace hepatic venous pressure gradient (HVPG) for portal hypertension evaluation and upper GI endoscopy for detecting varices.^19^ Adherence to these criteria may cause a delay in prophylaxis against variceal bleeding with beta-blockers in some patients. This difference in the validation of Baveno-VI criteria using noninvasive markers in different setups may be because of the many reasons, like LSM by TE may show frequent variability from position of the probe, body mass index, and the operator.^20^,^22^ For LSM-TE to be infallible, it should be done by an experienced person, defined as someone who has performed more than 100 examinations.^19^ LSM-TE may not be technically attainable in up to 20% of patients because of obesity. This problem has been partially overcome by using the XL probe in place of M probe, which can increase success rate to almost 85%.^23^ As Baveno-VI criteria were written when XL probe was not widely available, but now with its availability there is need to revise these recomendations.^24^ In our study we used XL in patients with BMI above 30 kg/m^2^. In addition, interpretation of TE should always be done with the clinical context and results of other tests. For example, past splenectomy may falsely normalize platelet count in the presence of severe portal hypertension and elevated ALT might increase it.^25^

Strength of our study is, this is the first study in this part of the world where we critically analyzed Baveno-VI recommendations of not doing screening Gastroscopies in cACLD patients. In our study we tried to minimize the misinterpretations of non-invasive markers of screening gastroscopy used before, e.g., transient elastography results by excluding patients with hepatic congestion, acute hepatitis, metabolic diseases, extrahepatic cholestasis and patients with history of splenectomy and being on interferon treatment, which were not considered before in some studies evaluated Baveno recommendations. We also highlighted that liver stiffness of 10 kPa which was used as a cut of for cACLD in Baveno-VI recommendations, does not necessarily indicate cirrhosis, this mainly indicates advance fibrosis which could be the reason of high negative predictive value in previous studies. ROC curve in our study suggested cutoff value of 11.5 kPa of LSM above which screening gastroscopy should be done.

### Limitation of the Study:

Our study had limitations of being single centered and cross-sectional study design so no temporal relation can be made. In future we will do multicentric studies with large sample size.

## CONCLUSION

Substantial number of VNT in cACLD patients were missed by strictly following Baveno-VI recommendations in our setup. Cut off value for screening gastroscopy found to be 11.5 kPa, may represent early cirrhosis complications in our setup. We recommend that all patients with cirrhosis with LSM of > 11.5 should be screened in our region provided they do not have any contraindication.

### Authors’ Contribution:

**FSA:** Conception & design.

**NB:** Analysis and interpretation of the data & drafting of the article.

**BFZ:** Critical revision of the article for important intellectual content, final approval of the article and is responsible for clinical integrity of the study.

**TR:** Drafting of the article.
